# Reassessing the Role of Eosinophils as a Biomarker in Chronic Obstructive Pulmonary Disease

**DOI:** 10.3390/jcm8070962

**Published:** 2019-07-02

**Authors:** Mariaenrica Tinè, Davide Biondini, Umberto Semenzato, Erica Bazzan, Manuel G. Cosio, Marina Saetta, Graziella Turato

**Affiliations:** 1 Department of Cardiac, Thoracic, Vascular Sciences and Public Health, University of Padova, 35128 Padova, Italy; 2 Respiratory Division, Meakins-Christie Laboratories, McGill University, Montreal, QC H4A3J1, Canada

**Keywords:** COPD, eosinophil, exacerbations

## Abstract

Blood eosinophils measurement, as proxy for tissue eosinophils, has become an important biomarker for exacerbation risk and response to inhaled corticosteroids (ICS) in Chronic Obstructive Pulmonary Disease (COPD). Its use to determine the pharmacological approach is recommended in the latest COPD guidelines. The potential role of blood eosinophils is mainly based on data derived from post-hoc and retrospective analyses that showed an association between increased blood eosinophils and risk of exacerbations, as well as mitigation of this risk with ICS. Yet other publications, including studies in real life COPD, do not confirm these assumptions. Moreover, anti-eosinophil therapy targeting interleukin (IL)-5 failed to reduce exacerbations in COPD patients with high blood eosinophils, which casts significant doubts on the role of eosinophils in COPD. Furthermore, a reduction of eosinophils might be harmful since COPD patients with relatively high eosinophils have better pulmonary function, better life quality, less infections and longer survival. These effects are probably linked to the role of eosinophils in the immune response against pathogens. In conclusion, in COPD, high blood eosinophils are widely used as a biomarker for exacerbation risk and response to ICS. However, much is yet to be learned about the reasons for the high eosinophil counts, their variations and their controversial effects on the fate of COPD patients.

## 1. Introduction

In the last few years, there has been a large number of publications about the possible role of blood eosinophils in both the prediction and treatment of chronic obstructive pulmonary disease (COPD) exacerbations. At the present time an increased number of eosinophils in blood, a possible biomarker for eosinophilic airway inflammation, has been used as a treatable trait in the management of COPD exacerbations [1,2]. The importance given to the role of eosinophils in COPD [3] stimulated the proposal of biological medications in order to diminish blood eosinophil numbers [4,5,6,7,8], but is there a solid base for these considerations?

## 2. Background

The potential role of blood eosinophils in COPD patients is based on data mainly derived from post-hoc and retrospective analyses of several large COPD clinical trials. Most of these studies, in which history of asthma was not systematically excluded, showed an association between an increased blood eosinophil count and risk of exacerbations, as well as mitigation of this risk with inhaled corticosteroids (ICS) [9,10,11,12,13,14,15,16,17,18]. A recent post-hoc analysis by Bafadhel et al. [19], in which subjects with a history of asthma were excluded, confirmed the use of the peripheral blood eosinophil count as a predictor of exacerbation risk and ICS treatment response in patients with COPD. The value of high blood eosinophils in predicting the exacerbation risk in COPD has also been examined in a recent large population study, which showed that the risk of both moderate and severe exacerbations was increased with higher eosinophil counts, but the effects were more pronounced in the severe exacerbation group. This might indicate that these patients may have a distinct inflammatory profile, making them more susceptible to develop severe events [10,20].

Taken together, these and other studies [21,22] support a relationship between blood eosinophil counts, COPD exacerbations and potential response to inhaled steroids in patients with COPD and history of exacerbations. These associations may indicate that eosinophils have a deleterious effect in COPD, however this does not necessarily imply a pathobiological role of eosinophils in the genesis of exacerbations.

The role of eosinophils as a biomarker to guide the response to inhaled steroids in COPD patients has been recently challenged [23,24,25,26]. In the ISOLDE study, the effect of ICS on the exacerbation rate was better in the COPD group with lower eosinophils than in the group with higher eosinophils [23]. Furthermore, treatment with LABA/LAMA (long-acting ß2-agonist/long-acting muscarinic antagonist) in the FLAME study was shown to be more effective than treatment with inhaled steroids in preventing COPD exacerbations regardless of the level, high or low, of blood eosinophils [24,25].

These controversial results might be due in part to the inclusion of patients with a history of asthma in many of these trials [9,12,15], which emphasizes the need for prospective long term studies on the possible role of blood eosinophils in the clinical outcomes of patients with pure COPD, i.e., patients without prior history of asthma. In fact, subjects with past history of asthma would still be likely to manifest many of the pathophysiological and inflammatory fingerprints of the original disease. Indeed, we have previously shown that patients with fixed airway obstruction and history of asthma maintain the typical pathological features of asthma (increased eosinophilic inflammation and thickened basal membrane), even when the fixed airway obstruction is well established [27].

For these reasons, it would be helpful that in future studies designed to assess the role of eosinophils in COPD, patients with COPD and previous history of asthma are analyzed as a separate group and compared with a COPD group without history of asthma.

## 3. Eosinophil Levels as a Biomarker

There is still no clear agreement at present on which level of blood eosinophilia should be used to guide preventive treatment with inhaled steroids. Recent publications recommend that for patients with one moderate exacerbation/year and blood eosinophils of 100–300 cells μL^−1^, inhaled steroids should be considered [28].

**Figure 1:**
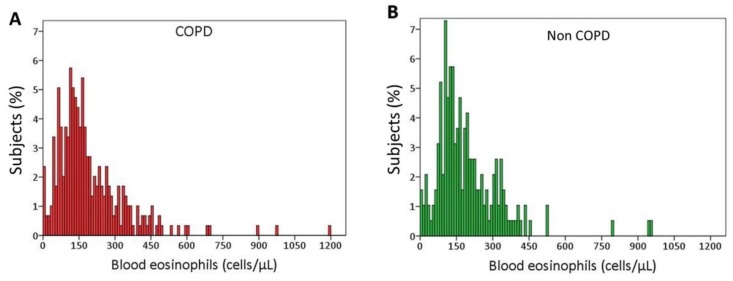
Distribution of blood eosinophils. Blood eosinophils distributions (in the blood sample obtained at patients recruitment) in a group of “real life” smokers followed for 5 years. (**A**) Smokers with chronic obstructive pulmonary disease (COPD) (*n* = 303) median, interquartile range: 156, 101–250 eosinophils μL^−1^. (**B**) Smokers without COPD (*n* = 209) median, interquartile range: 160, 107–256 eosinophils μL^−1^.

But what is the normal level of blood eosinophils in the normal population and in smokers with and without COPD? In a group of 512 “real life” smokers (i.e., smokers not selected for having prior exacerbations) with and without COPD and no history of asthma, blood eosinophils were measured yearly for 5 years [29]. Blood eosinophil values were similar in smokers with COPD (median, interquartile range: 156, 101–250 cells μL^−1^) and in those without COPD (160, 107–256 cells μL^−1^) (Figure 1), and these values were no different from those in normal populations [10]. Thus the recommendation of treating COPD patients based on a value of blood eosinophils above 100 cells μL^−1^ [3] would practically mean that the majority of COPD patients (about 75%) would be considered for treatment. In the study by Turato et al. [29], only a small percentage (3.3%) of smokers had blood eosinophil numbers exceeding the upper limit of normality (500 μL^−1^) [30], a percentage markedly lower than the 30% reported with asthma [31]. This suggests that COPD patients with blood eosinophils greater than 500 µL^−1^ at baseline might be asthmatics and possibly should be excluded from COPD trials, as done in the FLAME study where patients with a blood eosinophil count of >600 μL^−1^ were excluded [24].

A further important consideration when contemplating treatment choices based on a single blood eosinophil count, is that blood eosinophils are very variable, particularly when using higher (>300 cells µL^−1^) thresholds [2,29,32]. This is a significant issue not taken into consideration when assessing the role of a single blood eosinophil value in treatment decisions.

In spite of the previous observations it is presently believed, based on data derived mainly from post-hoc analyses of large COPD trials, that high (≥2%) blood eosinophils (equivalent to about ≥150 cells µL^−1^) are a useful biomarker for the prediction of frequent exacerbations and their prevention with the use of inhaled steroids [9,13,14,23]. However, it should be mentioned that most studies showing an association between frequency of COPD exacerbations, blood eosinophils and ICS response, have been done in patients selected for having a prior history of exacerbation. In studies in which patients were not selected for having previous exacerbations (real life COPD) [26,29,33], the exacerbation rate was not related to the number of blood eosinophils, a finding in agreement with previous publications where circulating eosinophils failed to predict future risk of COPD exacerbations [34,35].

We believe that studying “real life” patients is relevant, since it has been suggested that COPD patients selected for large research trials may represent only about 7% of patients in real life routine care [36], which raises the question of to what extent research trial data can be extrapolated to the entire COPD population.

## 4. Eosinophil Function in COPD

What could blood eosinophils in COPD tell us? Why should they be treated? Are they a reflection of the number of eosinophils in the airways and lung parenchyma as originally postulated? Studies comparing blood eosinophil numbers with numbers in large airways, small airways and parenchyma failed to show a correlation between blood and tissue eosinophils [29,37], thus we cannot assume that we are treating the possible tissue component, eosinophilia, in the prevention of exacerbations.

Even if tissue eosinophilia is not reflected by the blood values in COPD, it has been shown that eosinophils could be part of the inflammatory reaction seen in bronchial biopsies of exacerbated, but not in non-exacerbated COPD subjects with chronic bronchitis [38]. The airway eosinophilia in these cases was associated with an increase in RANTES (regulated upon activation normal T-cell expressed and secreted), a chemokine that recruits all inflammatory cells, including eosinophils, to sites of inflammation, however, at difference with asthma, was not associated to an increased expression of interleukin (IL)-5 [39,40]. These findings suggest that the mechanisms of eosinophils recruitment and their function in COPD exacerbations are different from those active in asthma. There is evidence that eosinophils have a function in the adaptive host response to viral pathogens [41] and it has been shown that the increase in sputum eosinophilia during exacerbations of COPD is related to viral loads [42]. These findings suggest that the mechanism of tissue eosinophilic inflammation in COPD might be a response to infection, mainly viral, recruited by RANTES and without involvement of IL-5. Thus, therapies geared to control IL-5 function in COPD could be misguided.

Interestingly blood eosinophils in COPD patients, even if well within normal limits, might have a paradoxical beneficial effect. It has been found that COPD subjects with eosinophil count ≥2% had a better pulmonary function, a better quality of life (St. George’s respiratory questionnaire) with fewer symptoms (modified medical research council questionnaire) and a lower BODE index [2], less comorbidities [43], and lesser rate of emphysema progression than subjects with eosinophil count <2% [2]. Furthermore, the risk of pneumonia in COPD patients, irrespective of the administration of inhaled steroids, was higher in patients with <2% than in those with ≥2% blood eosinophil count [44]. Moreover eosinopenia was associated with an increased risk of sepsis [45] and a worse outcome in patients presenting with an acute exacerbation of COPD [46,47]. These effects may be explained by the biological functions of eosinophils, that are first line players in the innate immune response against infections through their microbicidal function and their capability to act as antigen-presenting cells as well as to amplify the Th1 response [30,41,48].

**Figure 2:**
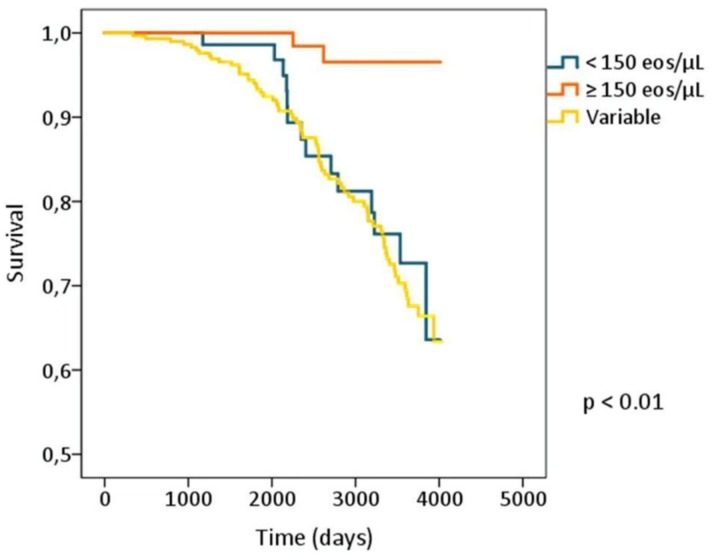
Survival in relation to blood eosinophil. Kaplan–Meier plots showing survival in smokers with blood eosinophils persistently <150 cells μL^−1^, persistently ≥150 cells μL^−1^ and variable (oscillating above and below 150 cells µL^−1^) over the 5 yearly blood samples. *p*-value < 0.01.

These beneficial effects of blood eosinophilia are in line with the observation [29,33] that subjects with persistently high blood eosinophils had a better survival rate than those with persistently low or variable blood eosinophils, as shown in Figure 2.Possibly, this finding is the result of the multiple beneficial effects reported in COPD patients with high eosinophil count, better lung function, less symptoms, less pneumonia, less comorbidities, which combined would allow for a better survival. 

Based on the reported effectiveness of inhaled steroids in exacerbations reduction, it was hypothesized that diminishing the numbers of blood eosinophils using biological medications might have been beneficial [5,6,7,8]. Treatment with IL-5 monoclonal antibody mepolizumab showed minimal efficacy in one of the two treated groups of eosinophilic COPD patients but not in the other [6]. Moreover, another targeted anti-eosinophil therapy, the anti-IL-5 receptor-α benralizumab, failed to reduce exacerbations in COPD patients with high blood eosinophils (>300 cells µl^−1^) and showed a tendency to increase exacerbations in patients with low eosinophils [7], a result confirmed by the recent GALATHEA and TERRANOVA trials [8]. Both these trials reported a substantial depletion of blood and sputum eosinophils that, unlike the results observed in benralizumab-treated asthma [49,50], did not reduce the annualized exacerbation rate among COPD patients [8]. Thus, reducing blood eosinophil counts, which is considered a treatable trait in COPD [1], does not improve patient’s outcome, which underlines the complexity of the disease and the multifactorial mechanisms of COPD exacerbations [20].

It is of interest that, in contrast with the studies that used biologicals, the use of inhaled steroids might reduce exacerbations in COPD subjects with high eosinophil counts but will not reduce the blood eosinophil numbers [51], which indicates that much has to be learned about the reasons for the high eosinophil counts, their variations and their controversial effects on the fate of COPD patients.

## 5. Conclusions

There is a growing consensus that the blood eosinophil count in COPD is a useful biomarker for the risk of exacerbations and response to ICS, however this is not supported by all trials. These discrepancies might be due to differences in the patient characteristics included in the studies. New clinical trials that would stratify patients according to blood eosinophil counts and compare COPD patients with and without previous history of asthma, might clarify this important issue. The variability of blood eosinophils, particularly when using higher (>300 µL^−1^) thresholds, is a significant issue when assessing the role of a single blood eosinophil value in treatment decisions. 

The fact that ICS reduce exacerbation risk without reducing blood eosinophil numbers while biological medications reduce eosinophil numbers without reducing exacerbations underscores the need for a better understanding of the role of eosinophil in COPD. It also suggests that the role of eosinophil in asthma is distinct from the role of eosinophil in COPD.

A final piece of information to be considered is that high blood eosinophils do not reflect lung tissue eosinophils, are not necessarily associated with worse outcomes and might even be beneficial.
